# Barriers and challenges in the process of including critically ill patients in clinical studies

**DOI:** 10.1186/s13049-020-00732-x

**Published:** 2020-06-08

**Authors:** Jørgen Dahlberg, Camilla Eriksen, Annette Robertsen, Sigrid Beitland

**Affiliations:** 1 Institute of Clinical Medicine, University of Oslo, Oslo, P.O.Box 1072 Blindern, 0316 Oslo, Norway; 2 Department of Anaesthesiology, Akershus University Hospital, Lørenskog, Norway; 3 Department of Anaesthesiology, Oslo University Hospital, Oslo, Norway; 4 Department of Research, Innovation and Education, Oslo University Hospital, Oslo, Norway

**Keywords:** Critical care, Informed consent, Intensive care, Mental capacity, Recruitment, Research ethics

## Abstract

**Background:**

Clinical research in severely ill or injured patients is required to improve healthcare but may be challenging to perform in practice. The aim of this study was to analyse barriers and challenges in the process of including critically ill patients in clinical studies.

**Methods:**

Data from critically ill patients considered for inclusion in an observational study of venous thromboembolism in Norway were analysed. This included quantitative and qualitative information from the screening log, consent forms and research notes.

**Results:**

Among 279 eligible critically ill patients, 204 (73%) were omitted from the study due to challenges and barriers in the inclusion process. Reasons for omission were categorised as practical in 133 (65%), medical in 31 (15%), and legal or ethical in 40 (20%) of the patients. Among 70 included patients, 29 (41%) consents were from patients and 41 (59%) from their next of kin. Several challenges were described herein; these included whether patients were competent to give consent, and which next of kin that should represent the patient. Furthermore, some included patients were unable to recall what they have consented, and some appeared unable to separate research from treatment.

**Conclusions:**

Barriers and challenges in the inclusion process led to the omission of near three out of four eligible patients. This analysis provided information about where the problem resides and may be solved. The majority of challenges among included patients were related to issues of autonomy and validity of consent.

**Trial registration:**

ClinicalTrials.gov (NCT03405766).

## Background

Clinical studies in severely ill or injured patients are essential to improve healthcare. There are, however, several barriers and challenges in actually including critically ill patients in clinical studies. Such obstacles may cause eligible patients fulfilling inclusion and exclusion criteria to be omitted from a study for various reasons. Among the included patients, there may be difficulties in retrieving valid consent for study participation. A next of kin consent is often provided in cases when patients are incompetent to give consent.

Previous studies have identified obstacles when performing research in critically ill patients at intensive care units (ICU) related to challenges in the recruitment process [1–4]. Some studies have described practical, ethical or legal challenges in obtaining informed consent [5–13]. Other studies have debated that such patients have compromised autonomy and reduced capacity to decide [14–18]. There is an ongoing debate on how to protect the patients in such research, and how to obtain a next of kin consent [19–28]. The legislation and clinical practice vary across the world, and a prior PubMed search did not disclose any resent Scandinavian research covering the overall barriers and challenges in the process of including critically ill patients in clinical studies.

The purpose of this study was to identify practical, medical, legal or ethical barriers and challenges in the process of including critically ill patients in the Norwegian Intensive Care Unit Dalteparin Effect (NORIDES) study. The primary aim was to identify and quantify barriers and challenges among eligible patients considered for inclusion and among included patients. A secondary aim was to report qualitative data on study investigators experiences during the inclusion process.

## Methods

### Study design and setting

The NORIDES study was a prospective, observational study of consecutive adult ICU patients admitted to Oslo University Hospital in Norway between December 2, 2012, and March 2, 2016. The aim was to investigate the effect of thromboprophylaxis with dalteparin in critically ill patients with and without acute kidney injury (AKI) treated with renal replacement therapy.

Patients included in the NORIDES study received standard treatment with additional Doppler ultrasound screening of veins to detect venous thromboembolism (VTE), and additional blood samples drawn from intravascular catheters for coagulation analyses. The main results of the NORIDES study describing the occurrence, risk factors and outcome of VTE is published [29], and additional results of coagulation tests are pending. In the NORIDES study, informed consent for participation was obtained from the patients or their next of kin in cases when patients were incompetent to give consent. When consent was provided from next of kin, patients were later informed wherever possible that they were included and had the right to withdraw from the study.

### Study population

Data from all patients considered for inclusion in the NORIDES study were included in this study; a detailed description of the study population is provided elsewhere [29].

### Data collection

Data were collected from the screening log, consent forms and research notes from study investigators in the NORIDES study.

Quantitative data were collected on the number of patients omitted from the NORIDES study, although they fulfilled the inclusion criteria and the reasons for such omission. We also collected data on the number of consents obtained from the patients or next of kin, and the number of oral and written consents.

Qualitative data on the study investigators experiences were collected. Notations were analysed and categorised in order to identify challenges and barriers experienced during the inclusion process and while collecting data from the patients.

## Results

### Barriers and challenges among patients considered for inclusion

**Fig. 1:**
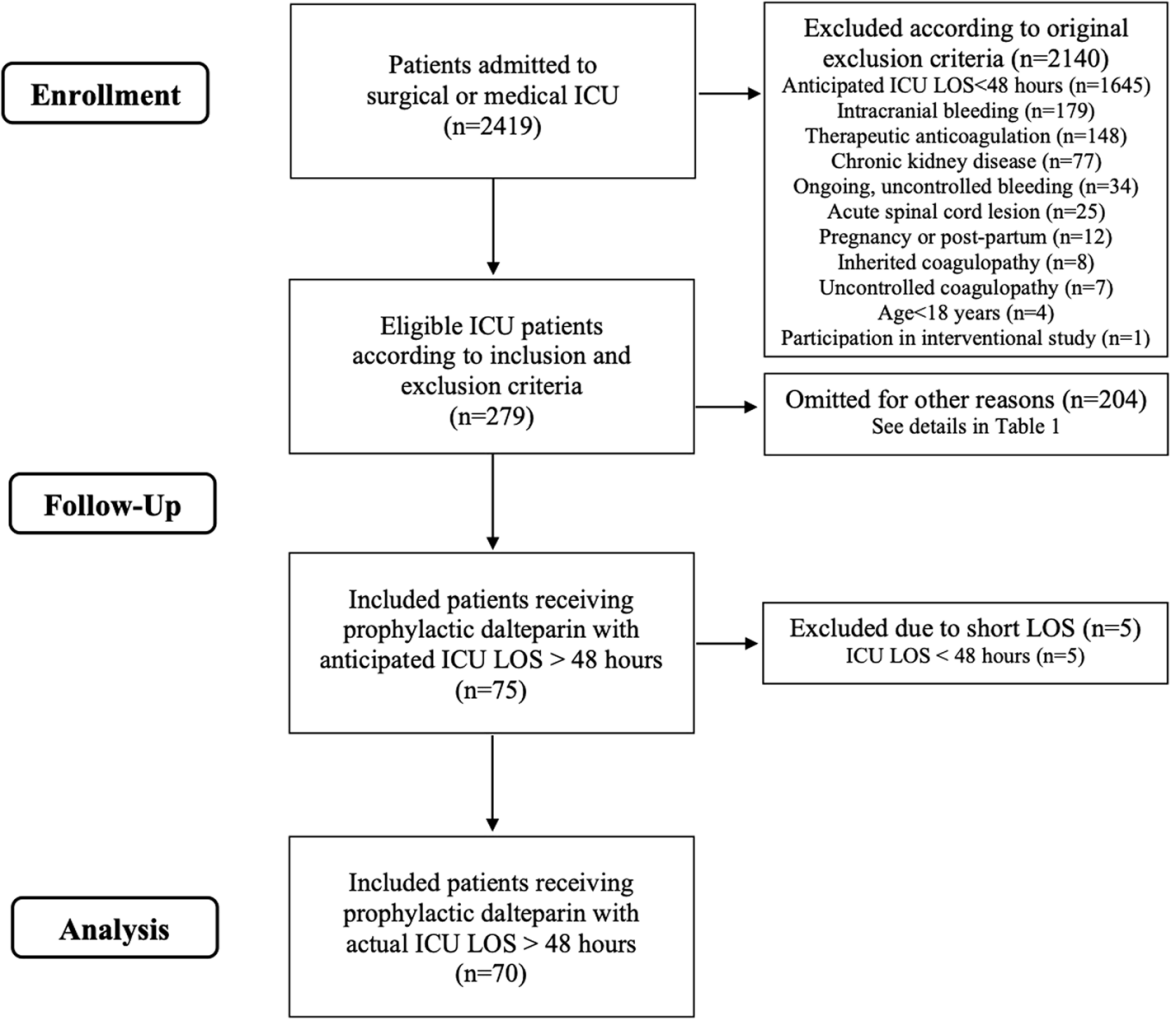
Flowchart of the study showing the process of patient enrolment, exclusion, omissions and inclusion; *ICU* intensive care unit, *LOS* length of stay, *n* number of patients

In the NORIDES study, 279 patients were eligible according to the inclusion and exclusion criteria predefined in the study protocol. Five of these were later excluded as predetermined because they were dispatched from the ICU within 48 h. Among eligible patients, 204 (73%) were omitted from the study due to challenges and barriers in the inclusion process (Fig. 1).

**Fig. 2:**
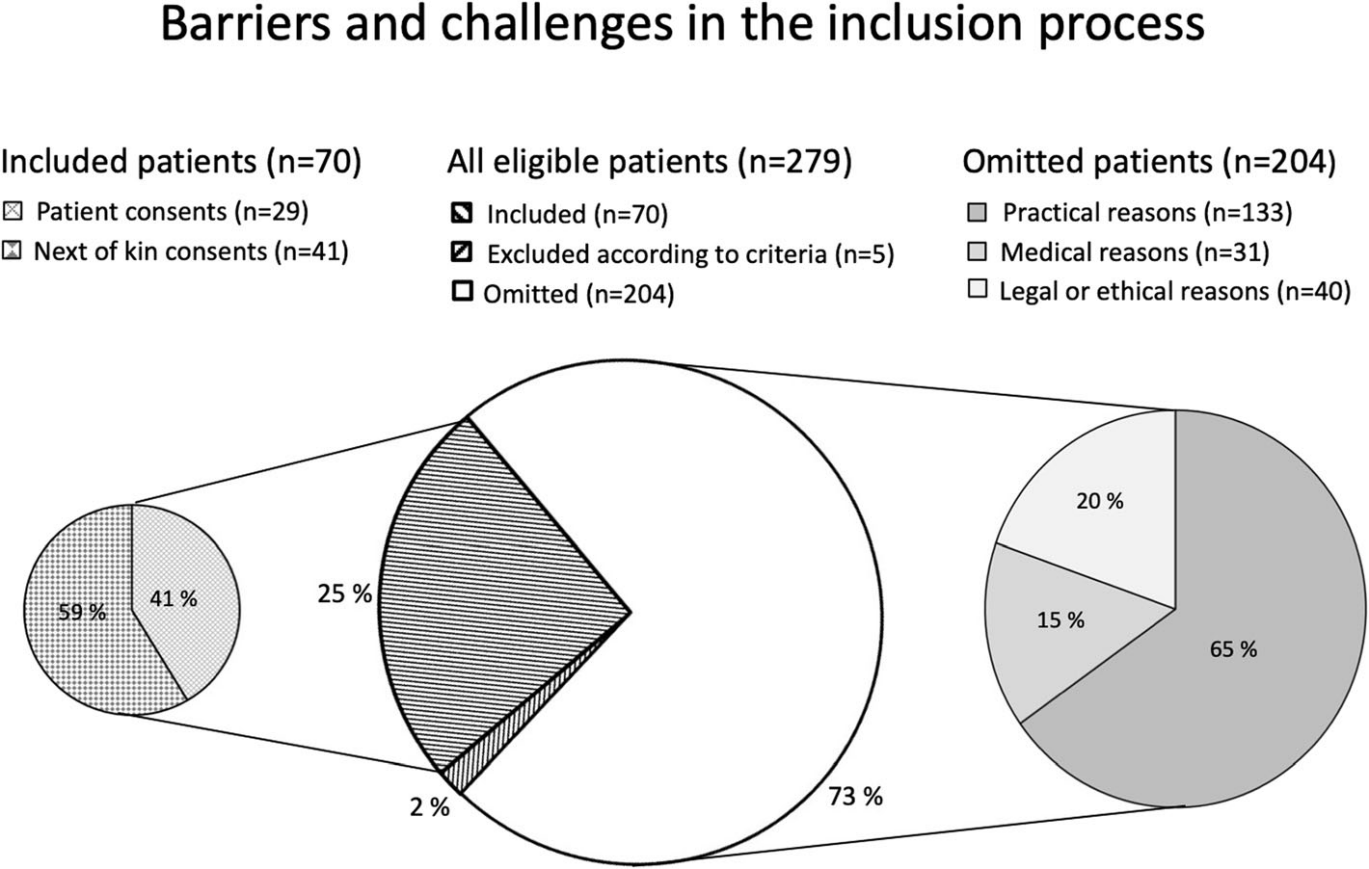
Overview of quantitative barriers and challenges in the process of including patients in clinical studies; *n* number of patients

The reasons for omission were categorised as practical in 133 (65%), medical in 31 (15%), and legal or ethical in 40 (20%) of the patients, respectively (Fig. 2).

Practical reasons for omission were lack of capacity to include, previous inclusions, communication barriers and too many patients without AKI already included. Medical issues causing omission from the study were low or high patient weight, plasmapheresis treatment and hygienic reasons. Legal or ethical reasons for omission were psychiatric conditions and end-of-life care (detailed description is presented in Table 1).

**Table 1:** Overview of patients omitted from the study and the reasons for omission

**Practical reasons (*n*** **= 133)**	
**Lack of capacity to include (*n*** **= 90)** Patients admitted to the ICU in periods where there were no study investigators available to include or adequately follow up patients were omitted, for instance, during holiday periods. **Previous inclusion (*n*** **= 7)** Patients already included in the study who were readmitted to the ICU were omitted to avoid double inclusion. **Communication barriers (*n*** **= 18)** Foreign language patients or next of kin where consent could not be acquired due to communication barriers in spoken and/or written communication were omitted. **Too many without acute kidney injury already included (*n*** **= 18)** The protocol for the NORIDES study required two evenly distributed patient groups with and without acute kidney injury, some patients without acute kidney injury were omitted to achieve even numbers in the groups. **Medical reasons (*n*** **= 31)**	
**Low or high patient weight (*n*** **= 11)** Patient weights were considered important for some of the outcomes of the study, patients below 50 kg or above 100 kg were therefore omitted as low or high patient weight were not exclusion criteria in the study protocol. **lasmapheresis treatment (*n*** **= 9)** Plasmapheresis treatment was considered important for some of the outcomes of the study; patients treated with plasmapheresis were therefore omitted from the study as it was not an exclusion criterion in the study protocol. **Hygienic reasons (*n*** **= 11)** The study involved an investigation with a Doppler ultrasound apparatus that could potentially transfer infectious diseases from one study participant to another; some patients were omitted to ensure infection prevention and control.	
**Legal or ethical reasons (*n*** **= 40)**	
**Psychiatric conditions (*n*** **= 21)** Patients admitted to the ICU following suicide attempts were omitted as it was not an exclusion criterion in the study protocol. However, the study personal considered that inclusion could add potential strain for the participants. **End-of-life care (*n*** **= 19)** Study personal omitted patients who were not expected to survive at admission or had treatment withdrawal during ICU stay. Both circumstances were not exclusion criteria in the study protocol. However, the study personal considered that inclusion could add potential strain to the patients or next of kin.	

Results are presented as numbers (n); *ICU* intensive care unit, *NORIDES study* Norwegian intensive care unit dalteparin effect study

### Barriers and challenges among included patients

Informed consent was provided for all 70 patients included in the NORIDES study. Among these consents, 29 (41%) were from patients, and 41 (59%) were from their next of kin.

Of the 29 consents from patients, 11 (38%) were oral, and 18 (62%) written (Fig. 2). In cases with oral consents, study investigator ensured a signature from a witness who was not part of the study. Several patients disclosed that they did not recognise their signature on the consent form directly after having signed.

In 41 cases, a valid consent could not be obtained from the patient, and consent was therefore obtained from their next of kin. Among these 41 patients, 39 were on mechanical ventilation during their ICU stay, and 13 remained on mechanical ventilation during the whole ICU stay. Ten of these 41 patients died at the ICU, and six were transferred to another ICU department.

**Fig. 3:**
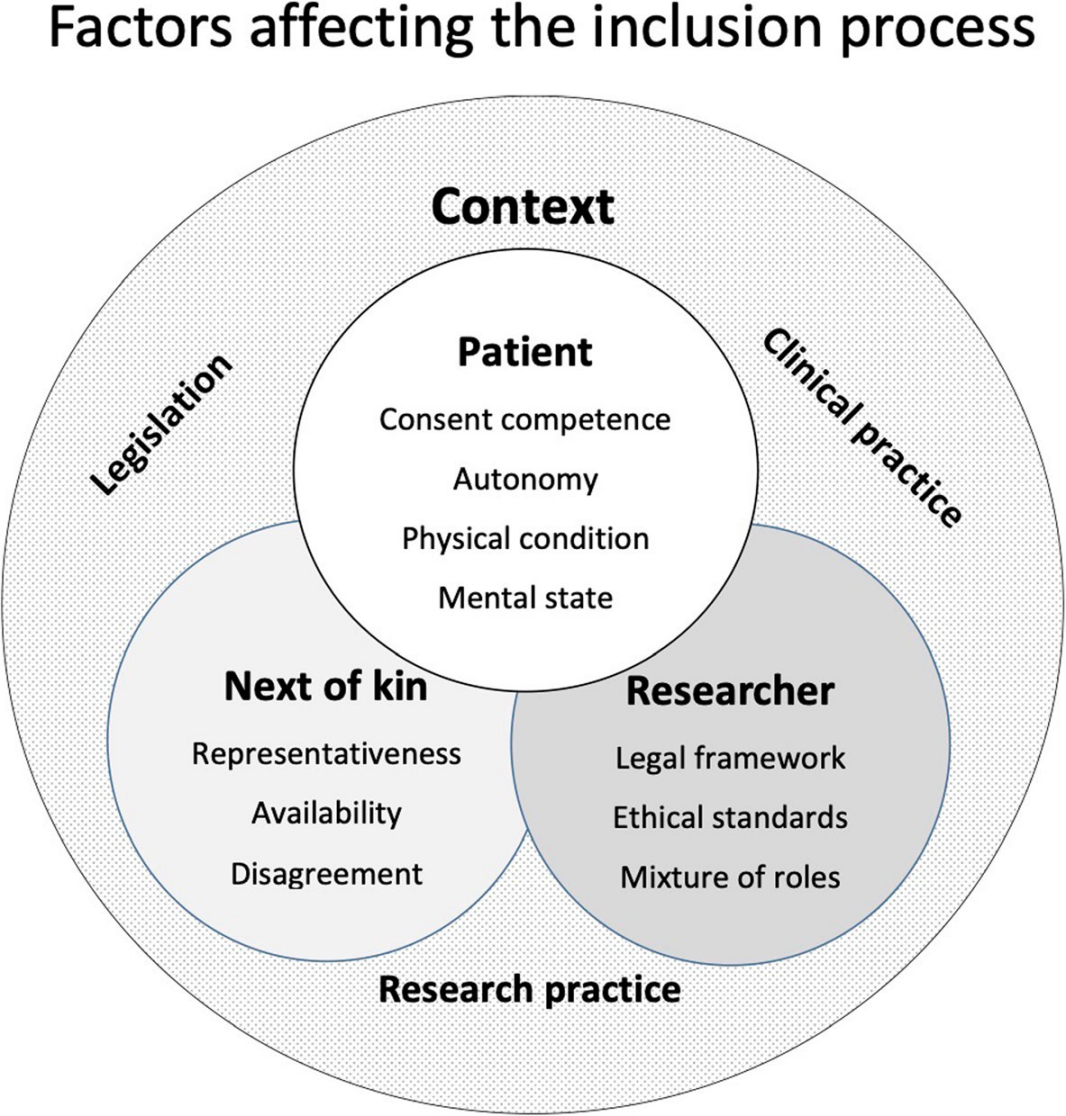
Overview of quantitative barriers and challenges in the process of including patients in clinical studies

The study investigators experienced several factors affecting the inclusion process (Fig. 3). They described that it was especially challenging to determine whether their severely ill patients were autonomous and competent to give consent. It appeared difficult to assess whether the patient actually “understood” what the information entailed due to their physical condition, treatment and mental state. Even though patients appeared to understand, appreciate, provide reasoning and express a choice, they still experienced that some of these patients were unable to recall being part of a study. Even though the information had been transferred thoroughly during inclusion, many patients did not remember that they had consented. When study investigators discovered this, they later sent a copy of study information and consent along with the patient at discharge from the ICU. Some patients were also unable to separate treatment from research, and when study investigator performed Doppler ultrasound of veins, some patients thought this procedure was part of their hospital treatment.

Another challenge reported by study investigators was difficulties in engaging next of kin. One challenge was the process of identifying which next of kin that should represent the patient, and how to solve any discrepancies in cases where several persons were involved as next of kin. In the NORIDES study, this was solved by obtaining consent from several next of kin in some cases. Another difficulty was to meet the next of kin, as they were often present at the hospital at evenings or weekends when study personnel were absent. Because of this, some consents from next of kin initially was obtained during telephone consultation, with later personal meeting and written consent.

Study investigators felt that it was problematic to mix their roles as treating physician and researcher for the same patients. They noted concerns that some patients may have consented to participation to keep the goodwill of the doctor. They also noted that it was difficult to assess autonomy and valid consents as many of the patient appeared to have reduced, but not necessarily absent, ability to understand.

## Discussion

This study revealed that near three out of four eligible patients fulfilling predefined inclusion, and exclusion criteria were omitted from the study due to barriers and challenges in the inclusion process. Such loss of participants in a study represents a possible source of attrition bias that alters the participants in a study [30]. Because omitted patients are not a random sample of eligible patients, this may affect study outcomes. The reasons for omissions in the present study were most often practical, followed by legal or ethical, and medical. The study revealed that some challenges might be avoided with better planning of the study or more resources available for study investigators, whereas other problems may be considered unavoidable.

Many factors do clinical research in critically ill patients challenging. The number of critically ill patients is limited, and patients are often acutely admitted outside working hours. The patients represent a heterogeneous group with variable baseline characteristics and acute organ dysfunctions. Because they have reduced body functions and require intensive treatment, the risk of study participation is high. In order to limit variability in the case-mix study and ensure the safety of patients, study investigators often use narrow inclusion criteria and broad exclusion criteria compared to researchers in safer elective settings.

As observed in the present study, researchers studying critically ill patients often end up with a relatively small proportion of admitted patients fulfilling eligibility criteria. Many patients in our study were omitted for reasons not necessarily specific to critically ill patients. These reasons may, however, have a more significant impact on this specific group of patients. For instance, since critically ill patients and their next of kin often are in a crisis, study investigators asking for consent should be able to understand the situation and act appropriately. Under these circumstances, obtaining consent should be performed by a person familiar with this setting. Another example may be hygienic considerations because critically ill patients are at high risk to become infected, hygiene will usually be prioritised higher than in most other clinical settings. Other reasons herein may be more numerous and specific for critically ill patients, such as ethical considerations in end-of-life care.

The practical reasons to omit patients from the present study could, to some extent, have been avoided. An issue that could be handled by extending the list of exclusion criteria in the study protocol was previous inclusion. Lack of capacity is here a significant contributor to omitting patients. Under ideal circumstances, this could be reduced with more resources available aiming at having available staff capable of obtaining informed consent 24 h a day, seven days a week or (if feasible) extending the timeframe of inclusion. The communication barrier could, to some extent, have been reduced by providing interpreter services, but this is often problematic with critically ill patients in an ICU setting. An alternative might be to add non-native speakers to the exclusion criteria in the study protocol. The challenge with too many without AKI already included seems unavoidable, unless researchers are willing to change the population of interest in the study or amending the inclusion system to in-roll in two groups.

All the medical reasons to omit patients in this study could have been identified as exclusion criteria in the initial protocol. However, with the complexity and heterogeneity of critical care patients, it may be challenging to identify all relevant exclusion criteria during the planning of a study, and several issues may be identified first after inclusion of patients has started.

The legal or ethical reasons to omit patients were pointed out as the most challenging by the study investigators. The reasoning for both the psychiatric and end-of-life care omission was the intent to lessen the burden for the patients and next of kin. This is a questionable assumption since participation could be conceived as a meaningful contribution to science for these patients [31–33].

In our study, no patients were omitted because they were unable to consent or that researchers were unable to determine consent competence. Challenges in obtaining consent were identified primarily as a problem regarding how to obtain valid consent for patients enrolled. The challenges herein were, therefore, questions regarding whether the patient still obtained personal autonomy by being competent to consent or not, alternatively by whom and how consent should be obtained from next of kin.

A general principle when obtaining patient consent is that the patient must be competent to give consent. This is often interpreted as a requirement for the patient to be able to understand, appreciate, reason and express a choice with regards to a specific question [15, 34]. Although some useful tools are developed to aid in these assessments, they only provide general guidelines that must be interpreted in a clinical setting [34–37]. The ability of critically ill patients to understand may vary depending on several fluctuating factors such as medication, tiredness and severity of illness, and the assessment of decision-making capacity may be challenging [9, 14].

There is Norwegian and international legislation regulating informed consent from the patient or next of kin in clinical research [38–41]. The current Norwegian legislation requires that the patient “clearly do not understand what the consent entails” in order to conclude on the lack of competence [42]. The assessment and decision on whether the patient is competent falls on the person responsible for obtaining the consent. Under this rule, the patient shall be treated as competent to consent if it is probable that the patient understands.

In our study, the researchers made their assessments of patients’ autonomy at the time of inclusion to the best of their ability. However, it remains unclear whether many of the enrolled patients had decision-making capacity at the time they consented. The fact that many were unable to recall having consented to be part of a study should probably be separated from consent competence [43]. Several options may be available to ensure that patients know that they have consented. One solution is to repeat the information several times to the patient; another is to provide information to their next of kin. A third possibility is to send written material about the study to the patients, for instance at discharge from the ICU or by postal mail after discharge.

Our study revealed that 38% of patient consents were oral, even though study investigators tried to obtain written consent whenever possible. Under Norwegian law, oral consent is considered as binding as written consent [38]. This may differ from other countries where specific requirements to written consent can pose a significant potential barrier on including critically ill patients in clinical studies. Since the burden of proof is more challenging in oral compared to written consent, it might be good practice to ensure a witness when obtaining oral consent. Our observation that many patients did not recognise their signature was probably because severe disease affected their handwriting capabilities or perception of the signature.

Less than half of the consents were directly from patients. The cause of this observation is for a large part due to the severity of illness in critically ill patients, which reduces their ability to be autonomous. Critically ill patients are especially challenging compared to other patient groups since they often have reduced or fluctuating consciousness, severe illness and high mortality rates [16, 17]. It is probable that these factors were present in our study, since many of the study participants were on ventilator treatment, and some died during ICU stay. The organisation of hospital treatment is also an explaining factor, as several patients were transferred from the university hospital to a local hospital before regaining consciousness and thereby, competence to give consent.

When the patients lack the competence to consent, representative consent may be obtained through the closest next of kin if researchers have approval for such practice [44]. As observed in our study, next of kin consents may also be challenging, even though this is partly regulated by law [45]. An obstacle is to decide which person that should be considered closest next of kin. This should be the person mentioned by the patient as their next of kin. If no such information is available, it should be the person who lives with the patient in a relationship resembling a marriage or partnership. If there is no such person, it should be the closest relative in the order of inheritance. An unsolved difficulty is how to handle disagreements in the question of consent between equally qualified representatives such as between parents or children. The regulations on appointing representation for patients without competence to consent may again differ significantly from other countries, thus providing this to be a variable barrier depending on the local jurisdiction. Clinicians and researchers should be aware that there might be a discrepancy between patients and surrogate opinion about treatment and research [22, 46–48].

An independent person who is neither part of the treating personnel nor engaged in the study may solve the challenges with patients and next of kin consent. In cases where researchers have approval for the use of such consent, this may prevent the misinterpretation of research being treatment, and add a third-party assessment. Such a person should seek the preferences of the patient and aim to conclude according to the probable wish of the patient.

The identified challenges in including critically ill patients in clinical studies raise concerns regarding how to ensure respect for the autonomy of patients [5–13]. Regarding this, researchers have to work within the local legal framework and practice shared decision-making. They also have an ethical obligation to act in the interest of the patient, and this may include several issues not covered by the legal requirements. Researchers should intend to optimise the decision-making capacity of patients; this entails to provide practical support and have adequate timing of the question of study participation.

Rules of informed consent are similar in observational and interventional studies and independent of the risk of study participation. Even though the legislation is similar, some argue that researchers have an ethical obligation to consider the risk profile of a study when obtaining consent and be especially cautious in high-risk studies [8]. In line with this, the mix of roles as researchers and clinicians for the same patients should be avoided whenever possible, because researchers have an interest in having patients included. However, such a separation of the roles may be difficult in clinical practice due to the lack of trained personal available, especially at small hospitals.

The described barriers and challenges in including critically ill patients in clinical research may have negative effects, including fewer study participants and thereby reduced statistical power. The main concern may be that omitted patients are not a random sample of eligible patients; this might affect study results because study participants are not representative of the population of interest. There may also be positive effects of omitting patients, for study quality, it may be necessary to avoid double inclusion and exclude patients with conditions that interfere with study outcomes. For patients, it is beneficial that they are protected against infectious diseases and having to consent under certain conditions as language barriers or end-of-life settings. Such omissions should, however, be properly described when reporting study results to ensure transparency.

This study has many limitations; including the single centre location, observational design and relatively low number of patients. Our findings could have been broadened by including data from other studies on critically ill patients as the challenges and barriers expectedly may vary depending on the specific study. We also refer to Norwegian laws and research, and it is clear that legislation and practice vary across the world. There is, therefore, a vast number of studies published touching on one or several of the challenges or barriers described by us pointing to other results. Strength of the study is that we included all challenges and barriers in the process of including critically ill patients in clinical studies. The study also provides qualitative data on the study investigators experiences during the inclusion process. It is important to have data from Scandinavian patients since they are previously not much described and might differ from other places.

## Conclusions

We observed that barriers and challenges in the process of including critically ill patients in research led to omissions of the majority of eligible patients from the study. This might be important information for clinicians and researchers, because such attrition bias may affect study outcomes. We further categorised these obstacles as practical, medical, legal or ethical, and discuss to which extent such obstacles are avoidable. The study revealed that most critically ill patients at ICU were unable to provide written, informed consent for study participation. Among patients who gave consent, we observed that the question of preserved autonomy and competence to consent was challenging. Even though patients appeared to be competent under the given rules, some were still unable to recall what they had consented, and some appeared unable to separate research from treatment. The use of next of kin as surrogate decision-makers provides additional challenges. Further studies on challenges and barriers in critical care research should be conducted in order to map out these important questions.

## Supplementary information

**Additional file 1.** Additional information.

## Data Availability

Data for this study is stored pursuant to the security requirements stated by the Regional Committee for Medical and Health Research Ethics at Oslo University Hospital and at the Medical Faculty, University of Oslo. The unidentified datasets used for analysis for the current study are available through the corresponding author on reasonable request.

## Abbreviations

AKI: Acute kidney injury
ICU: Intensive Care Unit
NORIDES: Norwegian Intensive Care Unit Dalteparin Effect study
VTE: Venous thromboembolism
