# Supplementary material for: Barriers and challenges in the process of including critically ill patients in clinical studies
Source: Scand J Trauma Resusc Emerg Med. 2020 Jun 8;28:51. doi: 10.1186/s13049-020-00732-x (PMC7276963; doi:10.1186/s13049-020-00732-x)
Supplement: Supplementary file 1 — Additional file 1. Additional information. [file 13049_2020_732_MOESM1_ESM.docx]

**Additional information**

**We performed a prior PubMed search which did not disclose any resent Scandinavian research covering the overall barriers and challenges in the process of including critically ill patients in clinical studies. We did this search using both controlled vocabulary (Medical Subject Headings) and title words. The search was restricted to English papers published in the last 20 years. On 14 February 2020, this yielded 151 articles. These were reviewed for relevance to our topic, and either by direct inclusion or indirect referral to other articles, this resulted in the majority of the references included.**

**The search strings were put together and restricted to last 20 years and to English language:
(Critical Care[Majr] OR Intensive Care Units[Majr] OR Critical Illness[Majr] OR Critical Care Nursing[Majr] OR Critical Care Outcomes[Majr] OR intensive care[ti] OR critical care[ti] OR critically ill[ti] OR critical illness[ti] OR serious illness[ti] OR seriously ill[ti] OR acute care[ti] OR ((intensive[ti] OR critical[ti]) AND (unit[ti] OR units[ti])) OR icu[ti]) AND ("Patient Selection"[Majr] OR "Ethics, Research"[Majr:NoExp] OR ((Research Subjects[Majr:NoExp] OR Researcher-Subject Relations[Majr] OR "Clinical Studies as Topic"[Majr] OR "Biomedical Research"[Majr:NoExp]) AND ("Patient Participation"[Mesh] OR "Informed Consent"[Mesh] OR "ethics" [Subheading]))) AND (study[Title] OR studies[Title] OR trial*[Title] OR research[Title]) AND "last 20 years"[PDat] AND English[lang]**

**The whole search strategy can be copied to PubMed:
https://www.ncbi.nlm.nih.gov/pubmed?otool=inouolib**
